# Novel Genetic Variants in *BAG3* and *TNNT2* in a Swedish Family with a History of Dilated Cardiomyopathy and Sudden Cardiac Death

**DOI:** 10.1007/s00246-017-1655-0

**Published:** 2017-07-01

**Authors:** Eva Fernlund, A. Wålinder Österberg, E. Kuchinskaya, M. Gustafsson, K. Jansson, C. Gunnarsson

**Affiliations:** 1 Department of Pediatrics, Department of Clinical Experimental Medicine, Linköping University, Linköping, Sweden; 2 Department of Clinical Genetics, Department of Clinical Experimental Medicine, Linköping University, Linköping, Sweden; 3 Department of Cardiology, Linköping University, Linköping, Sweden; 4 Department of Clinical Physiology, Linköping University, Linköping, Sweden; 5 Pediatric Heart Center, Lund University, S-22185 Lund, Sweden; 6 Centre for Rare Diseases in South East Region of Sweden, Linköping University, Linköping, Sweden

**Keywords:** Familial DCM, DCM, SCD, *BAG3*, *TNNT2*

## Abstract

Familial dilated cardiomyopathy is a rare cause of dilated cardiomyopathy (DCM), especially in childhood. Our aim was to describe the clinical course and the genetic variants in a family where the proband was a four-month-old infant presenting with respiratory problems due to DCM. In the family, there was a strong family history of DCM and sudden cardiac death in four generations. DNA was analyzed initially from the deceased girl using next-generation sequencing including 50 genes involved in cardiomyopathy. A cascade family screening was performed in the family after identification of the *TNNT2* and the *BAG3* variants in the proband. The first-degree relatives underwent clinical examination including biochemistry panel, cardiac ultrasound, Holter ECG, exercise stress test, and targeted genetic testing. The index patient presented with advanced DCM. After a severe clinical course, the baby had external left ventricular assist as a bridge to heart transplantation. 1.5 months after transplantation, the baby suffered sudden cardiac death (SCD) despite maximal treatment in the pediatric intensive care unit. The patient was shown to carry two heterozygous genetic variants in the *TNNT2* gene [*TNNT2* c.518G>A(p.Arg173Gln)] and *BAG3* [*BAG3* c.785C>T(p.Ala262Val)]. Two of the screened individuals (two females) appeared to carry both the familial variants. All the individuals carrying the *TNNT2* variant presented with DCM, the two adult patients had mild or moderate symptoms of heart failure and reported palpitations but no syncope or presyncopal attacks prior to the genetic diagnosis. The female carriers of *TNNT2* and *BAG3* variants had more advanced DCM. In the family history, there were three additional cases of SCD due to DCM, diagnosed by autopsy, but no genetic analysis was possible in these cases. Our findings suggest that the variants in *TNNT2* and *BAG3* are associated with a high propensity to life-threatening cardiomyopathy presenting from childhood and young adulthood.

## Introduction

Cardiomyopathies are defined as myocardial disorders in which the heart is structurally and functionally abnormal; in the absence of coronary artery disease, valvular heart disease, hypertension, or congenital heart disease sufficient to cause the observed myocardial abnormality [1].

In pediatric cardiomyopathy registries, the incidence of DCM have been reported to be 1/140 000–1/170 000 [2, 3], the clinical course is often severe [2]. Dilated cardiomyopathy is also the most frequent underlying diagnosis leading to pediatric heart transplantation [3].

At young ages, DCM may be caused by congenital heart disease, coronary anomalies, arrhythmias, myocarditis, myopathies, or metabolic cardiomyopathy, although some cases remain idiopathic [2, 4, 5]. Familial dilated cardiomyopathy (FDC) is a rare cause of DCM, especially in childhood [2].

Dilated Cardiomyopathy is characterized primarily by left ventricular dilatation and impaired systolic function and is one of the leading causes of heart failure with high morbidity and mortality. Pediatric DCM is defined by the presence of left ventricular end diastolic diameter (>2SD, in relation to body surface area), fractional shortening less than 25% (>2SD), and ejection fraction (EF) less than 45% (>2SD), excluding any known cause of myocardial disease [4, 6]. The disease occurs even in pediatric cases and the incidence among children have been shown to be higher in infants (<1 year old) compared to patients ages 1–18 years [7, 8] and is higher among boys than girls [9]. Familial dilated cardiomyopathy is identified in 20–48% of cases with DCM [4], less common in pediatric DCM [2]. If the pedigree can reveal more than one individual with DCM are denoted as FDC [4, 10].

The genetic spectra have involved variants in over 50 genes of diverse ontology, most of which encoding sarcomeric or sarcomeric-associated proteins [11]. Most variants lead to an autosomal dominant pattern of inheritance; however, a minority is associated with recessive, X-linked or maternal mitochondrial forms. Penetrance may be incomplete (the proportion of mutation-positive individuals who show the phenotype) and disease expression (the degree of severity among known affected, mutation-positive individuals) is variable. The wide spectra of the expression of the disease in the same family can make the clinical follow-up difficult.

In the published guidelines from 2009 and 2011, genetic evaluation is recommended in families with DCM as cardiovascular screening of at-risk family members and consideration of genetic testing in individuals with DCM [11]. Guidelines from 2011 recommended LMNA and SCN5A genetic testing for individuals with DCM and significant conduction system disease or premature, unexpected sudden cardiac death in a family [12]. However, these guidelines were published before some important reports that pointed out new genes of interest for developing DCM, for example TTN [13].

Our aim is to present a family with a history of SCD and dilated cardiomyopathy, the presentation of heart failure in infants and to discuss the clinical relevance of genetic testing.

## Clinical Description

The index patient is a girl, born after a normal gestation. The first months in life were happy. At three months of age, there was onset of recurrent crying attacks especially at night, failure to thrive, and a mild transient respiratory infection occurring at the same time. Feeding difficulties started at the age of 3.5 months, after some days increasing breathing problems.

**Fig. 1:**
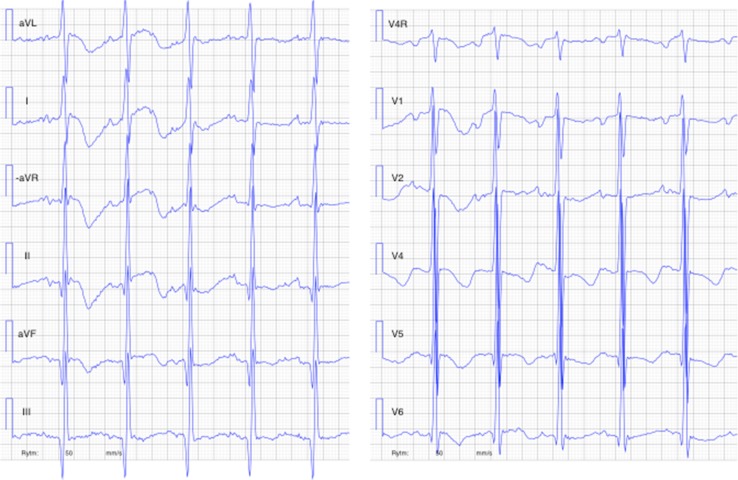
The first ECG in the proband (V:3) at the time of presentation, 4 months old, showing a picture of electrocardiographic hypertrophy and ST-depression over left ventricle

**Fig. 2:**
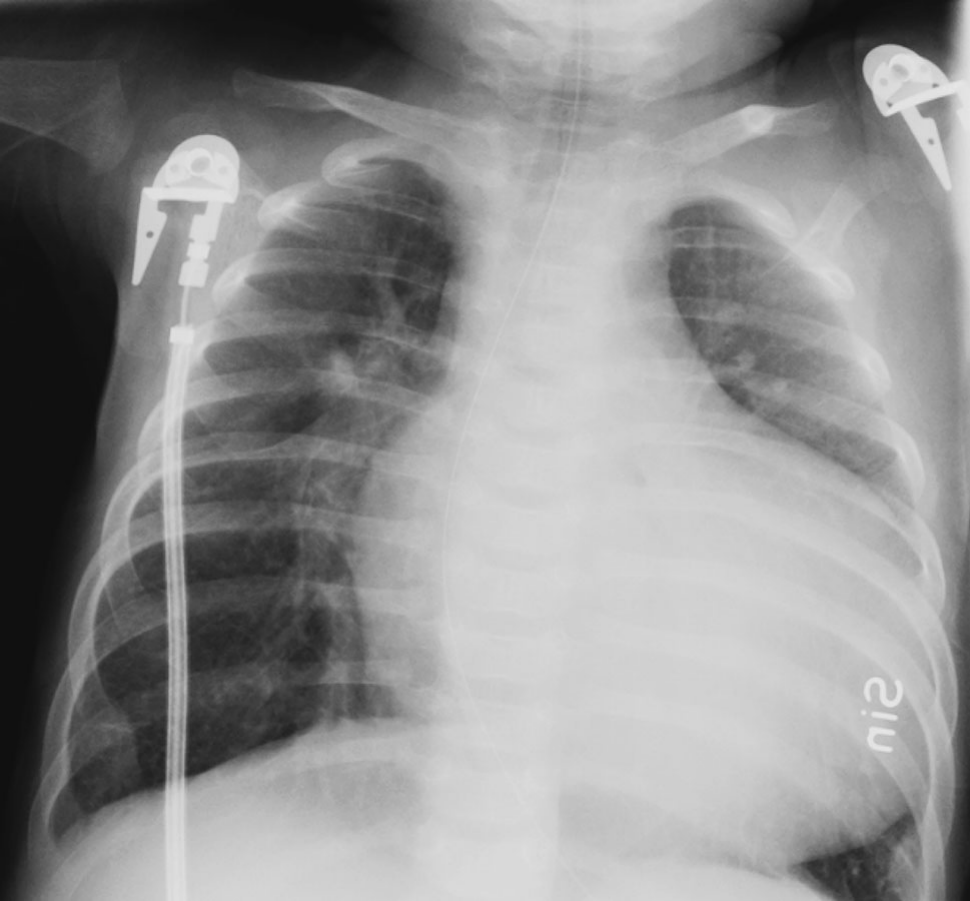
The chest X-ray at the time of presentation in the 4 month old proband, showing the severely enlarged heart

**Fig. 3:**
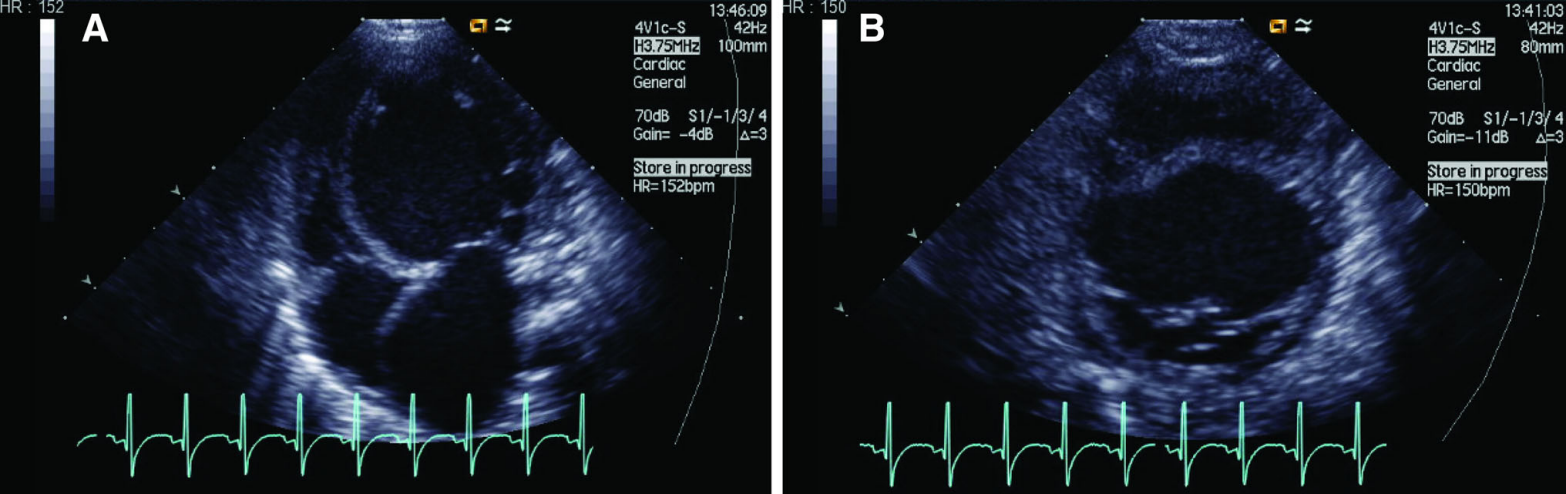
The echocardiogram at the time of presentation in the proband.** a** Apical four-chamber view showing the enlarged left ventricle.** b** Short-axis view showing the enlarged left chamber

The girl was admitted to the hospital at 4 months of age with severe breathing problems. She was pale, in prechock, arterial oxygen saturation 70%, pH 7.14, base excess-12. An initial treatment with furosemide and CPAP (Continuous Positive Airway pressure) was successful. Standard 12-lead ECG was severely pathologic showing sinus tachycardia, enlarged P-waves, generally enlarged amplitudes, and QTc prolongation, Fig. 1. Chest X-ray revealed a magnificent enlargement of the heart, Fig. 2. Echocardiography revealed a dilated cardiomyopathy and poor left ventricular contractility, Fig. 3. The girl was transferred to the pediatric cardiology center where additional cardiac examinations were performed.

Echocardiography at presentation showed a severely enlarged left ventricle, LVID 47 mm (upper limit 26 mm relative to body surface area), fractional shortening 12%, Fig. 3. The left atrium was enlarged, moderate mitral regurgitation was present, but no structural mitral valve abnormalities were observed. The aortic valve was found to be bicuspid, the annulus 8 mm (*z*-score −1.6 SD) but no measureable stenosis in the aortic valve or aortic arch was present. The girl underwent a cardiac catheterization that excluded ALCAPA (anomalous left coronary artery from pulmonary artery) as well as significant aortic stenosis. During the procedure, there was need for cardiac resuscitation due to a circulatory collapse. After Levosimendan infusion and some days of stabilization, a cardiac biopsy could be performed that showed a picture of lymphocytic myocarditis.

The pharmacologic treatment consisted of captopril, carvedilol, warfarin, furosemide, and spironolactone. Because of the severe left ventricular impairment, immunoglobulin and interferon A were also given to the patient. Despite intensive pharmacological treatment due to heart failure and repeated Levosimendan infusions, the ventricular function declined over time.

There was a second circulatory collapse by the age of 6.5 months that ended in implantation of a left ventricular assist device, that later was changed to the Berlin Heart EXCOR^®^ Pediatric Ventricular Assist Device (VAD). A cardiac transplantation was performed at 9.5 months of age. The follow-up biopsies were initially normal as well as the left ventricular contractility assessed by echocardiography. One month post-transplant, the echo showed a good systolic function but mild progressive septal hypertrophy, decreasing tissue Doppler velocities (TDI), but unremarkable cardiac biopsy. 1.5 months post-transplant, an impressive septal hypertrophy was present, further drop in TDI and diastolic function but good systolic function were noted. Optimal medication was administrated to the patient in the intensive care unit but despite treatment, a final irreversible circulatory collapse occurred. Postmortem examination revealed biventricular hypertrophy with subendocardial fibrosis and diffuse myocardial damage. In the postmortem, histochemical analysis showed lymphocytic humoral rejection.

The family history revealed a history of sudden cardiac death (SCD) due to DCM. The girls’ maternal grandfather, his brother and their father, died unexpected in the early 40s, autopsy did show enlarged left ventricle in these relatives.

## Methods

In this family, there was a strong family history of DCM, why a cascade family screening was initiated. It included clinical evaluation, control of medical history, review of medical records, physical examination, biochemistry panel, 12-lead electrocardiogram, echocardiography, Holter ECG, exercise stress test, and blood samples for genetic evaluation were obtained after informed consent was provided.

DNA extraction from whole blood samples was performed using either EZ1 (Oiagen) or Prepito (Techtum). DNA concentration and quality was determined using NanoDrop spectrophotometer. Samples with A260/A280 ratios between 1.8 and 2.0, and A260/A230 ratios above 1.5 were accepted for further sequencing.

DNA was analyzed and the following genes were included: *ABCC9, ACTC1, ACTN2, ANKRD1, BAG3, CASQ2, CAV3, CRYAB, CSRP3, CTF1, DES, DSG2, DSP, DTNA, EMD, FHL2, GATAD1, GLA, JUP, LAMA4, LAMP2, LDB3, LMNA, MYBPC3, MYH6, MYH7, MYL2, MYL3, MYLK2, MYOZ2, NEBL, NEXN, PKP2, PLN, PRKAG2, RBM20, RYR2, SCN5A, SGCD, TAZ, TCAP, TMEM43, TMPO, TNNC1, TNNI3, TNNT2, TPM1, TTN, TTR,* and *VCL*. Variants were reported according to HGVS nomenclature (www.hgvs.org/mutnomen). This test was performed by oligonucleotide-based target capture (Sureselect, Agilent) followed by next-generation sequencing (Illumina HiSeq 2000). All clinically significant and novel variants were confirmed by independent Sanger sequencing.

Upon completion of basic clinical and genetic evaluation, the affected and at-risk family members underwent a clinical follow-up program.

## Results

**Fig. 4:**
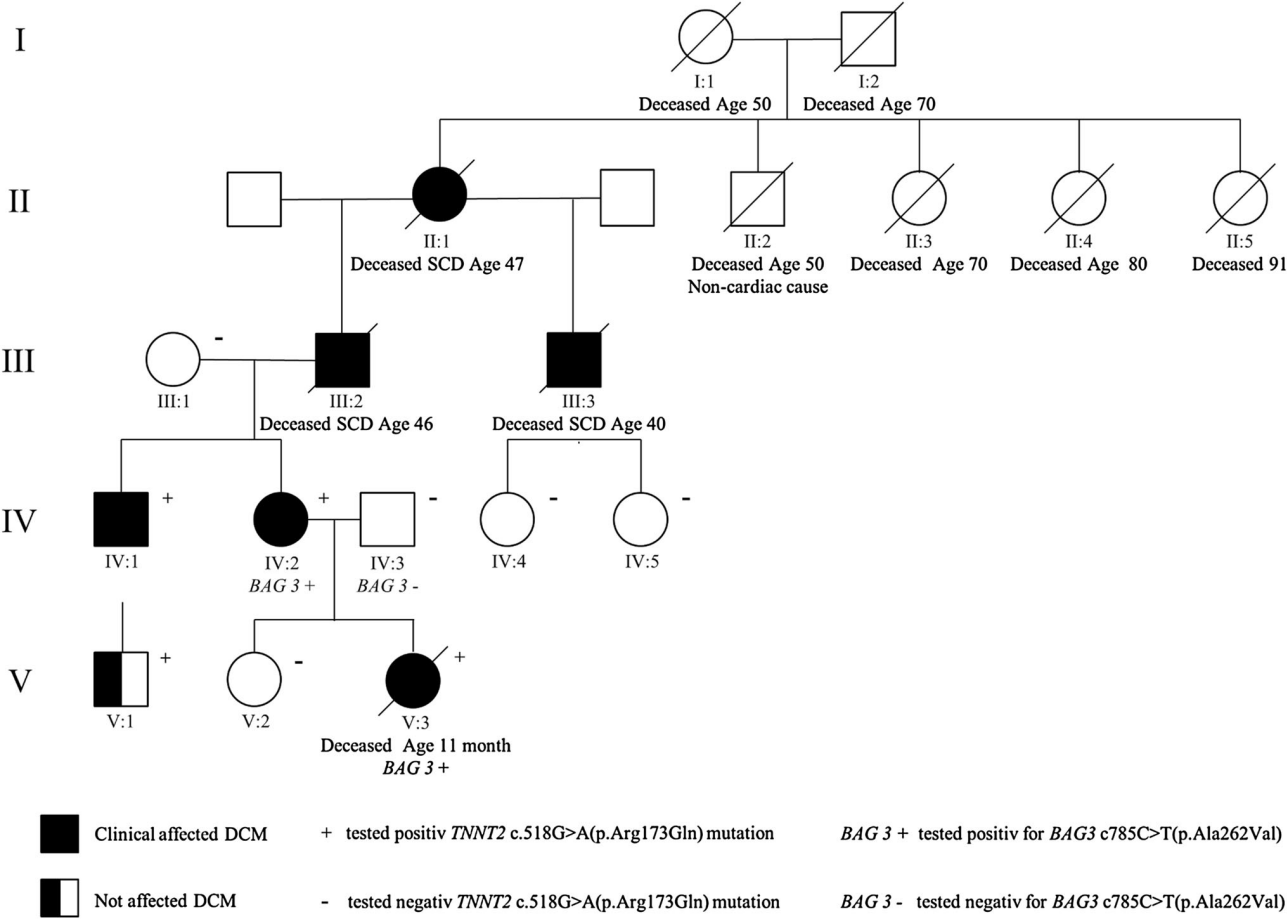
The pedigree of the family. The proband V:3 developed DCM at 4 months of age, V:1 developed clinical DCM at the age of 18 years, and IV:1 and IV:2 at the early thirties. In the older generations, SCD due to DCM in their early forties (II:1, III:2, III:3)

At early stage, a pedigree was performed in the present family, Fig. 4, where DCM could be found in four generations in the present family. The index patient (V:3) presented with advanced DCM and severe heart failure. After a severe clinical course with decline of cardiac function, there was a need of external left ventricular assist as a bridge to heart transplantation. A successful heart transplant was performed, but 1.5 months after transplantation the baby suffered sudden cardiac death (SCD) despite maximal treatment in the pediatric intensive care unit. The genetic evaluation of the index patient showed two heterozygous genetic variants in *TNNT2* c.518G>A(p.Arg173Gln) and *BAG3* c.785C>T(p.Ala262Val). These genetic variants were shown to be inherited from the maternal family.

At the same time, as the diagnosis of the index patient, the girls’ maternal uncle (IV:1), 35 years old at that time, was diagnosed with DCM. Due to non-specific chest discomfort, left bundle branch block on ECG, and the history of early cardiac death in the family, the uncle underwent echocardiography that revealed a moderately dilated left ventricle (LVID 66 mm), mild mitral regurgitation, and reduced ejection fraction (EF 35%). Usual workup including radionuclide myocardial perfusion imaging excluded ischemic heart disease as a cause of his cardiac dysfunction. After initiation of pharmacological treatment with beta-blockers and ACE inhibitors, the patient became asymptomatic and NT-proBNP and Troponin T levels returned to normal range. However, serial echocardiographic exams demonstrated a slow decline in cardiac function, ejection fraction declined to 25%, and he was provided with a primary prophylactic CRT-D device. After CRT-D implantation, there have been improvement of systolic function to EF 50% and reduction of left ventricular dilatation (LVID 62 mm), reduced mitral regurgitation and left atria is normal in size at the last follow-up. There are indirect signs of diastolic dysfunction though normal E/e´values. In the ICD-arrhythmia recordings (CRT-D device), there have been several short non-sustained VT but no anti-tachycardia therapies or ICD-discharges have been delivered to the patient.

The son of the uncle (V:1) was found to be carrier of the *TNNT2* variant at 14 years, and recently, at the age of 18 years, he has been found to have DCM-diagnosis with moderately reduced EF why ACE-I therapy was initiated.

The mother (IV:2) of the index patient was at the time 34 years of age, complained of shortness of breath and palpitations especially during physical activity, but she had performed two successful pregnancies without symptoms. She went through a thoughtful investigation and was found to have a dilated left ventricle 60 mm and a reduced ejection fraction 25%. There were no signs of clinical heart failure, arrhythmia, or coronary artery disease. After initiation of pharmacological treatment with beta-blockers and ACE inhibitors, she responded well, is asymptomatic and her levels of NT-proBNP are normal. Her left ventricle inner diastolic diameter (LVID) was 56 mm and EF was 45% at the last follow-up. She received a primary prophylactic ICD. No anti-tachycardia therapies or ICD-discharges have been delivered to this patient.

Subsequently, two of the screened individuals were found to be carriers of the *TNNT2* and *BAG3* variants. The family members with the variant in *TNNT2* all showed echocardiographic DCM, while the family members with *TNNT2* and *BAG3* variants were found to have more advanced DCM. The index patient suffered fatal DCM and the adult case had moderate reduction of left ventricular systolic function. During follow-up, the adult case has shown decline of left ventricular function measured by ejection fraction, and has received a primary prophylactic ICD. The two cases with sole *TNNT2* variant have got the diagnosis of DCM at young age and one of them has received a CRT-D device. In the family history, there were three additional cases of SCD due to DCM (III:2, III:3 and II:1), unfortunately, no genetic test could be performed in these historic cases.

## Discussion

Recurrent breathing problems in an infant along with failure to thrive should always lead to suspicion of an underlying heart disease. As clinical-physical examination does not always give correct clues to distinguishing breathing problems due to heart disease from obstructive bronchitis, chest X-ray is of great importance in these cases, directing the patient to echocardiography in the first line diagnostics. The diagnosis of DCM in an infant is crucial, as it is usually accompanied with high morbidity and risk of mortality; therefore, these pediatric cases of DCM most often require admittance to pediatric cardiology center for further diagnostic interventions and advanced treatment.

In this actual case, the index patient suffered a severe DCM with a complicated and fatal clinical course despite optimal treatment. The proband was shown to carry two heterozygous genetic variants in *TNNT2* c.518G>A(p.Arg173Gln) and *BAG3* c.785C>T(p.Ala262Val), a combination that has not been described earlier. In this family, there was a striking family history of DCM and the cascade screening revealed additional cases of clinical and echocardiographic DCM.

This family shows an autosomal dominant inheritance of familial dilated cardiomyopathy in four generations, although with different clinical penetrance and expression among the family members. In this particular family, the phenotype ranges from early onset of lethal DCM in the young proband, to later onset of the disease in the adult family members, according to the pedigree, Fig. 4.

Variants in the *TNNT2* gene have earlier been reported in different cardiomyopathies, hypertrophic, non-compaction [14] and dilated cardiomyopathy [15]. The *TNNT2* gene is placed on chromosome 1q32 and coding for a sarcomere-related protein. *TNNT2* c.518G>A has previously been associated with DCM and shown to segregate with disease in five affected members of the same family, including one sudden cardiac death. Van Acker et al. reported this variant to co-segregate with disease in five individuals from three generations of a family affected by DCM [16]. In addition, Hernandez Del Rincon reported in a poster session [17] that they found this variant in a case of sudden cardiac death in Spain (19th International Association of Forensic Sciences World Meeting). The brief poster abstract does not contain additional phenotype data for the deceased, such as whether DCM was detected, although it mentions that a complete autopsy was performed. The variant in *TNNT2* identified in this family has been described once earlier, in a prenatal-onset disease [16]. The family described by Van Acker et al. shows a great phenotype difference among family members carrying the mutation. One can speculate if the variants in *TNNT2* alone are responsible for the aggressive phenotype in the young girl in our family or the prenatal case in the family presented by Van Acker et al.; is it possible to explain this by another modulating variants in another part of the genome or did the infectious disease in the index patient act as a trigger event for the underlying hereditary cardiomyopathy?

In this family, we also detected a variant of unclear significance in *BAG3* in the index patient. Recently, Norton et al. have performed genome-wide studies of copy number variation and exome sequencing and identified rare variants in *BAG3* as a cause of DCM [18]. *BAG3* is highly expressed in the heart and variants in *BAG3* are known to cause myofibrillar myopathy with restrictive or hypertrophic cardiomyopathy [19].

The Ala262Val variant in *BAG3* has not been identified in large European American and African American populations by NHLBI Exome Sequencing Project (http://evs.gs.washington.edu/EVS), though it may occur in other populations. Alanine (Ala) at position 262 is not conserved in mammals or evolutionarily distant species and horse carries a valine (Val; this variant), suggesting that this change may be tolerated. Additional computational analyses (biochemical amino acid properties, AlignGVGD, PolyPhen2, and SIFT) also suggest that the Ala262Val variant may not impact the protein, though this information is not predictive enough to rule out pathogenicity. A different variant (Ala262Thr) at the same position has been reported in two brothers with familial DCM [18], though the significance of this variant is also unclear. In summary, additional information is needed to fully assess the clinical significance of the Ala262Val variant in *BAG3*.

Two of the screened individuals appeared to be carriers of both the familial variants in this family, four of six screened individuals carried the *TNNT2*-variant with or without development to DCM, showing the importance of a careful family history, structured family screening based on systematic pedigree analysis, clinical examination, and genetic testing in suspicion of an inherited cardiac disease, which can dramatically alter the future for the other affected family members or the individuals at risk for the disease.

## Conclusion

We report a novel familial genetic variant causing FDC due to a *TNNT2* variant with a possible modifier in the *BAG3* variant. Unlike previous descriptions, this new variant is associated with a malignant cardiac phenotype associated with the early onset of DCM, presenting as breathing problems due to severe heart failure associated with a severe clinical course ending in lethal complications in the 11 month-old baby, and a family history of DCM or sudden death in a four-generation family. This study also illustrates the importance of structured family screening and the yield of genetic testing in suspicion of an inherited cardiac disease.

In conclusion, our findings suggest that the *TNNT2* variant especially in combination with the *BAG3* variant is associated with a high propensity to life-threatening cardiomyopathy presenting from childhood and young adulthood.
